# Identification of Heparin-Binding EGF-Like Growth Factor (HB-EGF) as a Biomarker for Lysophosphatidic Acid Receptor Type 1 (LPA_1_) Activation in Human Breast and Prostate Cancers

**DOI:** 10.1371/journal.pone.0097771

**Published:** 2014-05-14

**Authors:** Marion David, Debashish Sahay, Florence Mege, Françoise Descotes, Raphaël Leblanc, Johnny Ribeiro, Philippe Clézardin, Olivier Peyruchaud

**Affiliations:** 1 INSERM, U1037, Toulouse, France; 2 Institut Claudius Régaud, Toulouse France; 3 INSERM, U1033, Lyon, France; 4 Université Claude Bernard Lyon 1, Villeurbanne, France; 5 Faculté de Médecine Lyon Est, Lyon, France; 6 Hôpital Edouard Herriot, Hospices Civils de Lyon, Lyon, France; 7 Centre Hospitalier Lyon Sud, Hospices Civils de Lyon, Pierre Bénite, France; Columbia University, United States of America

## Abstract

Lysophosphatidic acid (LPA) is a natural bioactive lipid with growth factor-like functions due to activation of a series of six G protein-coupled receptors (LPA_1–6_). LPA receptor type 1 (LPA_1_) signaling influences the pathophysiology of many diseases including cancer, obesity, rheumatoid arthritis, as well as lung, liver and kidney fibrosis. Therefore, LPA_1_ is an attractive therapeutic target. However, most mammalian cells co-express multiple LPA receptors whose co-activation impairs the validation of target inhibition in patients because of missing LPA receptor-specific biomarkers. LPA_1_ is known to induce IL-6 and IL-8 secretion, as also do LPA_2_ and LPA_3_. In this work, we first determined the LPA induced early-gene expression profile in three unrelated human cancer cell lines expressing different patterns of LPA receptors (PC3: LPA_1,2,3,6_; MDA-MB-231: LPA_1,2_; MCF-7: LPA_2,6_). Among the set of genes upregulated by LPA only in LPA_1_-expressing cells, we validated by QPCR and ELISA that upregulation of heparin-binding EGF-like growth factor (HB-EGF) was inhibited by LPA_1–3_ antagonists (Ki16425, Debio0719). Upregulation and downregulation of HB-EGF mRNA was confirmed *in vitro* in human MDA-B02 breast cancer cells stably overexpressing LPA_1_ (MDA-B02/LPA_1_) and downregulated for LPA_1_ (MDA-B02/shLPA_1_), respectively. At a clinical level, we quantified the expression of LPA_1_ and HB-EGF by QPCR in primary tumors of a cohort of 234 breast cancer patients and found a significantly higher expression of HB-EGF in breast tumors expressing high levels of LPA_1_. We also generated human xenograph prostate tumors in mice injected with PC3 cells and found that a five-day treatment with Ki16425 significantly decreased both HB-EGF mRNA expression at the primary tumor site and circulating human HB-EGF concentrations in serum. All together our results demonstrate that HB-EGF is a new and relevant biomarker with potentially high value in quantifying LPA_1_ activation state in patients receiving anti-LPA_1_ therapies.

## Introduction

Lysophosphatidic acid (LPA) is a natural bioactive lipid involved in multiple physiological processes [1]–[7]. LPA is a potent signaling molecule with pleiotropic biological actions that through genomic and/or nongenomic activities induces cell proliferation, survival, motility, cytoskeletal rearrangement, and differentiation [8]. LPA activates a series of six different G protein-coupled receptors (LPA receptors [LPA_1–6_]) [9], [10] that are distributed into two subfamilies. LPA_1,_ LPA_2_ and LPA_3_ form the Endothelial Differentiation Gene (EDG) subfamily and LPA_4,_ LPA_5_ and LPA_6_ form a subfamily closely related to purinergic receptors. All LPA receptors share intracellular signaling pathways dependent on heterotrimeric G protein subtypes such as Gα_i_ (LPA_1–4,6_), Gα_12/13_ (LPA_1–2,4–6_), Gα_q_ (LPA_1–5_), and Gα_S_ (LPA_4,6_) [11], [12] that upon activation potentially lead to redundant, synergistic or even opposite effects on cell biology. Most eukaryotic cells co-express multiple LPA receptors. Therefore, pleiotropic activities of LPA are likely the consequence of co-activation signals mediated by multiple receptors.

LPA_1_ is the most ubiquitous of all LPA receptors in organs and tissues both in human and mouse [13]. *Lpar1* ^−/−^ mice revealed that LPA_1_ signaling influences the pathological process of many diseases. These animals are partially protected against bleomycin-induced lung fibrosis [14] and *Lpar1* ^−/−^ mice immunized with type II collagen do not develop arthritis [15]. Additionally, *Lpar1* ^−/−^ mice are resistant to neuropathic pain induced by partial nerve ligation, and both allodynia and hyperalgesia induced in by intrathecal injection of LPA is totally abolished in these animals [16]. LPA_1_ is highly involved in cancer development. LPA_1_ has pro-oncogenic and pro-metastatic activities [17] and recent developments of anti-LPA_1_ pharmacological drugs showed that targeting this receptor is a therapeutic option for metastasis suppression [18]–[20].

Therefore, LPA_1_ is an attractive target in multiple clinical situations. However, current developments of LPA receptor inhibitors are based on the use of LPA receptor-null cells, such as the rat hepathoma RH7777 cells transfected to express recombinant LPA receptor specific subtypes, and measuring the capacity of the drugs inhibiting LPA-induced Ca^2+^ release in the cytoplasm and GTPγS binding to the plasma membrane [21], [22]. However, no specific LPA receptor biomarker has been defined so far, which impairs validation of specificity and efficacy of these drugs in more complex systems such as in cells expressing multiple subtypes of LPA receptors, as well as *in vivo* both in animals and humans. LPA_1_ was shown to induce the secretion of IL-6 and IL-8 in ovarian and breast cancer cells [23], [24]. However, LPA_2_ and LPA_3_ also induce the secretions of these cytokines [23], [24]. Renal cells from *Lpar1* ^−/−^ mice showed impaired expression of CTGF indicating a close relationship between LPA_1_ activity and CTGF expression [25]. However, TGF-β also controls CTGF expression [26]. Thus, the specificity of CTGF expression through LPA_1_ activation requires further demonstration.

Here, we identified a set of genes specifically upregulated through the activation of LPA_1_ based on transcriptomic analyses of nongenetically manipulated tumor cells selected on the bases of their different cancer of origin and on their distinct expression panel of LPA receptors. Among those genes, we demonstrated *in vitro* and *in vivo* that heparin-binding EGF-like growth factor (HB-EGF) is a new specific biomarker for LPA_1_ activity in human breast and prostate cancers. Our findings revealed that HB-EGF is a potential new biomarker that will be useful to monitor the LPA_1_ activation state in patients receiving anti-LPA_1_ therapies.

## Experimental Procedures

### Ethic statement

The mice used in our study were handled according to the rules of Décret N° 87–848 du 19/10/1987, Paris. The experimental protocol was reviewed and approved by the Institutional Animal Care and Use Committee of the Université Claude Bernard Lyon-1 (Lyon, France). Studies were routinely inspected by the attending veterinarian to ensure continued compliance with the proposed protocols. Male BALB/C nude mice, 4 weeks of age, were housed under barrier conditions in laminar flow isolated hoods. Autoclaved water and mouse chow were provided ad libitum. Animals bearing tumor xenografts were carefully monitored for established signs of distress and discomfort and were humanely euthanized when these were confirmed. Studies involving human primary breast tumors were performed according to the principles embodied in the Declaration of Helsinki. Tissue biopsies were obtained as part of surgical treatments for the hormone receptor content determination. Remaining samples were included anonymously in this study. All human experiments were approved by the Experimental Review Board from the Laennec School of Medicine that waived the need for consent.

### Drugs and reagents

Lysophosphatidic acid (LPA, Oleoyl C18:1) was obtained from Avanti Polar Lipids. The competitive inhibitors of LPA signaling pathways dependent on LPA_1_ and LPA_3_ receptors, Ki16425 was obtained from Cayman and Debio0719 was obtained from Debiopharm SA.

### Cell lines

Human cancer cell lines (MDA-MB-231, MCF-7 and PC3) were obtained from the American Type Culture Collection. Characteristics of MDA-B02/GFP-βGal breast cancer cells were described previously [27]. Characteristics of MDA-B02/LPA1 and MDA-B02/shLPA1 breast cancer cells were described previously [18], [28]. All cell lines were cultured in complete media, DMEM medium (Invitrogen), 10% (v/v) fetal bovine serum (FBS, Perbio) and 1% penicillin/streptomycin (Invitrogen), at 37°C in a 5% CO_2_ incubator.

### Reverse transcription and polymerase chain reaction (RT-PCR)

Total RNA from cells and mouse xenograph tumors were extracted using Nucleospin RNAII kit (Macherey-Nagel) and cDNA were synthesized using iScript cDNA Synthesis kit (Biorad). The cDNAs were amplified by PCR for 35 cycles consisting of 10 s of denaturation at 95°C, 15 s of annealing at 67°C, and 10 s of extension at 72°C with the following specific PCR primers: HB-EGF-F (5′-GGACCCATGTCTTCGGAAAT-3′) and HB-EGF-R (5′-CCCATGACACCTCTCTCCAT-3′) for HB-EGF; LPA1-F (5′-TGGCATTAAAAATTTTACAAAAACA-3′) and LPA1-R (5′-AATAGTTACAACATGGGAATGG-3′) for LPA_1_; LPA2-F (5′-CGCTCAGCCTGGTCAAGACT-3′) and LPA2-R (5′-TTGCAGGACTCACAGCCTAAAC-3′) for LPA2; LPA3-F (5′-GGAGGACACCCATGAAGCTA-3′) and LPA3-R (5′-GGAACCACCTTTTCACATGC-3′) for LPA3; L32-F (5′- CAAGGAGCTGGAAGTGCTGC-3′) and L32-R (5′- CAGCTCTTTCCACGATGGC-3′) for L32. Expression of mRNAs were quantified by real-time quantitative RT-PCR in an Eppendorf Mastercycler RealPlex (Invitrogen) using the SYBR Green PCR kit (Finnzymes). Quantifications of target genes were normalized to corresponding L32 RNA values.

### Affymetrix analysis

Large-scale mRNA expression profiling was done on MDA-MB-231, MCF-7 and PC3 cells untreated and treated with LPA (1 µM) for 45 min. Total RNAs were purified as described above. Labeled cRNA probes from two independent replicates were hybridized to GeneChip Human Genome U1033 plus 2.0 (Affymetrix). Data were analyzed using Affymetyrix expression console (v.1.1) software by ProfilExpert (Lyon, France). Mean fluorescent signals of duplicated probe sets were calculated. All probe sets with more than 1.3-fold increase in presence of LPA defined LPA-dependent upregulated genes. The data discussed in this publication have been deposited in NCBI's Gene Expression Omnibus [29] and are accessible through GEO Series accession number GSE56265 (http://www.ncbi.nlm.nih.gov/geo/query/acc.cgi?acc=GSE56265).

### Microarray correlation analysis

Publicly available gene expression data for Prostate Tumor (GSE2109); Lung Tumor (GSE43580) and Colon Tumor (GSE21510) were obtained. Log2 tranformed values for HB-EGF and LPA1 expression were extracted from these database using R2 genomics analysis and visualization platform. Scatter plots were constructed. Analysis of correlation and computation of linear regression of the data were performed using Prism v5.0b (GraphPad Software, Inc.). *P* values <0.05 were considered significant.

### Patients and tumor characteristics

Patients were selected according to the following criteria: primary breast tumor without inflammatory feature, no previous treatment. Patients tumors were provide by three medical centers (Centre Hospitalier Régional Annecy, Chirurgie Oncologique Centre Hospitalier Universitaire Lyon-Sud, and Clinique Mutualiste Saint Etienne, France) in which patients were included between October 1994 and October 2001. Breast cancer tissue biopsies were obtained by surgery, selected by the pathologist and immediately stored in liquid nitrogen until processing. The biopsies were pulverized using a Mikro-Dismembrator (B. Braun Biotech International, Melsungen, Germany) and total RNAs were extracted using TRI Reagent (Sigma). To remove any genomic DNA contamination, total RNAs were treated with RNAse-free DNAse I and purified using RNeasy microcolumns (Qiagen). Quality of RNAs was verified using an Agilent Bioanalyser 2100 (Agilent).

### Animal studies

Tumor xenograph experiments were performed using PC3 cells. Cells were suspended at a density of 10^6^ cells in 100 µl of PBS and inoculated subcutaneously into the flank of male BALB/C nude mice at 4 weeks of age (Charles River). Tumor size was assessed by external measurement of the length (L) and width (W) of the tumor using a Vernier caliper. Tumor volume (TV; expressed in mm^3^) was calculated using the following equation: TV = (LxW^2^)/2. Thirty-five days post-tumor cell injection animals were treated daily for 5 days with subcutaneous injection of Ki16425 (25 mg/kg). Then animals were sacrificed. At that time the serum of each animal was collected for human HB-EGF quantification by ELISA (RayBiotech Inc) and PC3 xenograph tumors were submitted to total RNA extraction as described above.

### Statistical analysis

Data were analyzed with the GraphPad Prism v5.0c software. Differences between groups were determined by one-way ANOVA followed by Bonferroni post-test and single comparisons were carried out using two-sided unpaired t-Test. Clinical multiple group comparisons were performed using Kruskall-Wallis followed Dunn's post-test. *P*<0.05 was considered significant.

## Results

### Determination of LPA_1_-specific early genes upregulated by LPA

**Figure 1:**
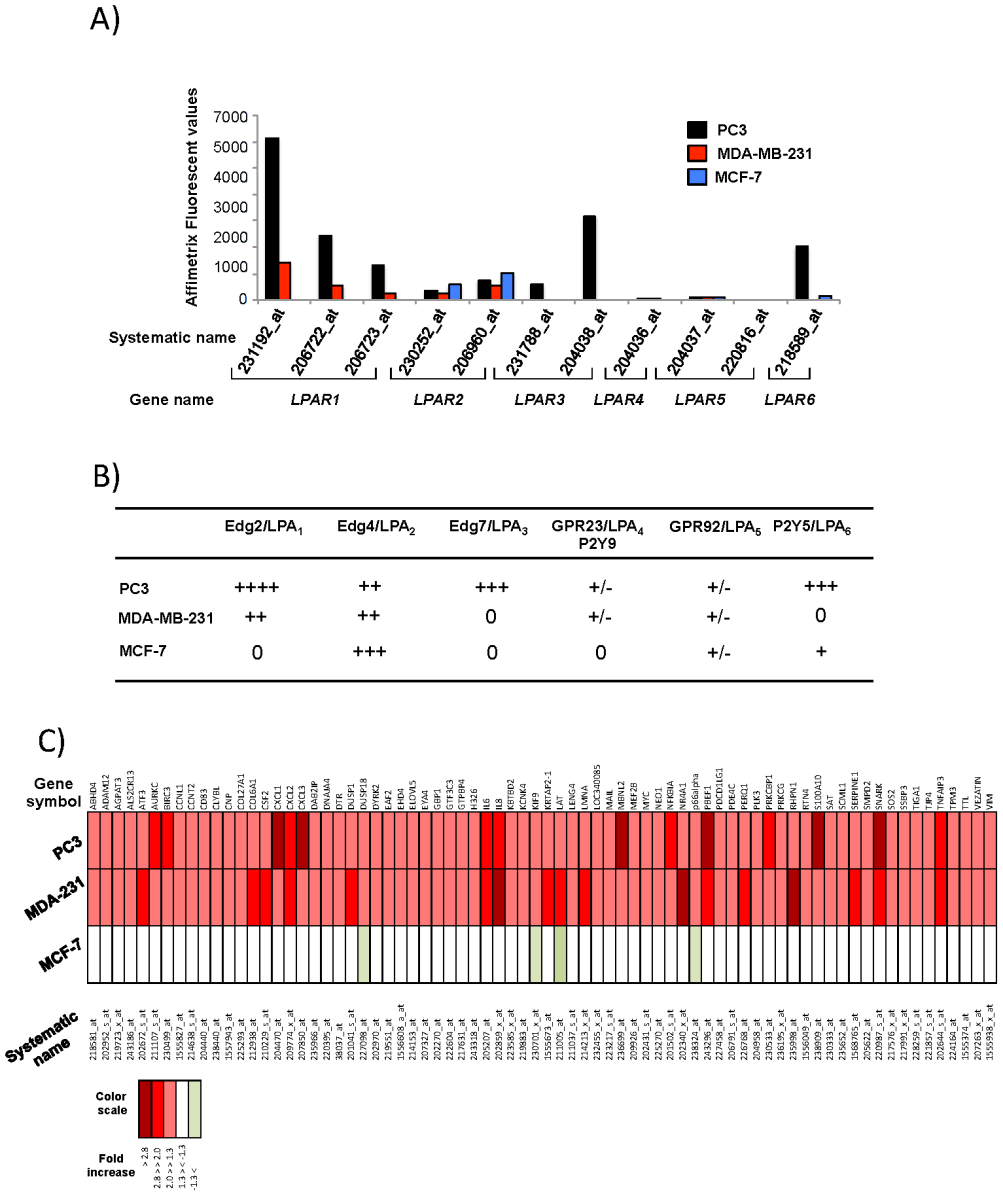
Determination of LPA_1_-specific early genes upregulated by LPA. (A) Fluorescent values (Y-axis) of Affimetrix probe sets corresponding to each LPA receptor (X-axis) generated using total RNAs isolated from PC3, MDA-MB-231 and MCF-7 cells. (B) Relative expression levels of LPA receptors in PC3, MDA-MB-231 and MCF-7 cells extrapolated from Affimetrix fluorescent values presented in A). (C) Heat map of genes significantly upregulated in both MDA-MB-231 (MDA-231) and PC3 cells and not in MCF-7 cells stimulated by LPA (1 µM) for 45 min. Color scale corresponds to fold increase.

LPA_1_ is the most ubiquitous LPA receptor in mammalian tissues. As a consequence, its expression in cells and tissues is frequently associated with other LPA receptors. Therefore, to identify LPA_1_ activated genes independently of cell backgrounds we defined a strategy based on the use of nongenetically modified cell lines expressing different patterns of LPA receptors. In addition, to avoid activation of cell type-specific genes we selected the cell lines from two different human cancers of origin, breast (MDA-MB-231 and MCF-7) and prostate (PC3). For breast cancer cells we used two different types of cells, an estrogen positive cell line (MCF-7) and a triple negative cell line (MDA-MB-231). Hama and colleagues in 2004 showed that PC3 cells express LPA_1_, LPA_2_ and LPA_3_, MDA-MB-231 cells express at least LPA_1_ and LPA_2_, and MCF-7 cells do not express LPA_1_ nor LPA_3_ but LPA_2_ [30]. These cells were subjected to total RNA extractions that were labeled and cRNA were probed on Affimetrix GeneChip Human Genome U1033 plus 2.0**.** In order to define the LPA receptor expression profiles in each cell line, we first extracted the fluorescent values obtained for each Affimetrix probe set corresponding to LPA receptor genes (Figure 1A). Our results confirmed that the cell lines used in our study expressed differently LPA_1_, LPA_2_ and LPA_3_ receptors (Figure 1B). Moreover, we could determine the complete LPA receptor patterns in PC3 cells (LPA_1_, LPA_2_, LPA_3_ and LPA_6_), MDA-MB-231 cells (LPA_1_ and LPA_2_), and MCF-7 cells (LPA_2_ and LPA_6_). This result indicated that LPA_2_ was expressed in all three cell lines and that LPA_1_ was the only receptor communally represented in PC3 and MDA-MB-231 cells (Figure 1B). Then we observed that LPA (1 µM) stimulation for 45 min upregulated 253 probe sets in PC3 cells, 283 in MDA-MB-231 cells, and 400 in MCF-7 cells and downregulated 465 probe sets in PC3 cells, 470 in MDA-MB-231 cells, and 332 in MCF-7 cells (data not shown). Taking advantage that LPA_1_ was expressed only in PC3 and MDA-MB-231 cells and not in MCF-7 cells, we identified a list of 74 distinct genes that were upregulated downstream activation of LPA_1_-specific signaling pathways (Figure 1C).

### Expression of HB-EGF is mediated through functional LPA_1_ in vitro

**Figure 2:**
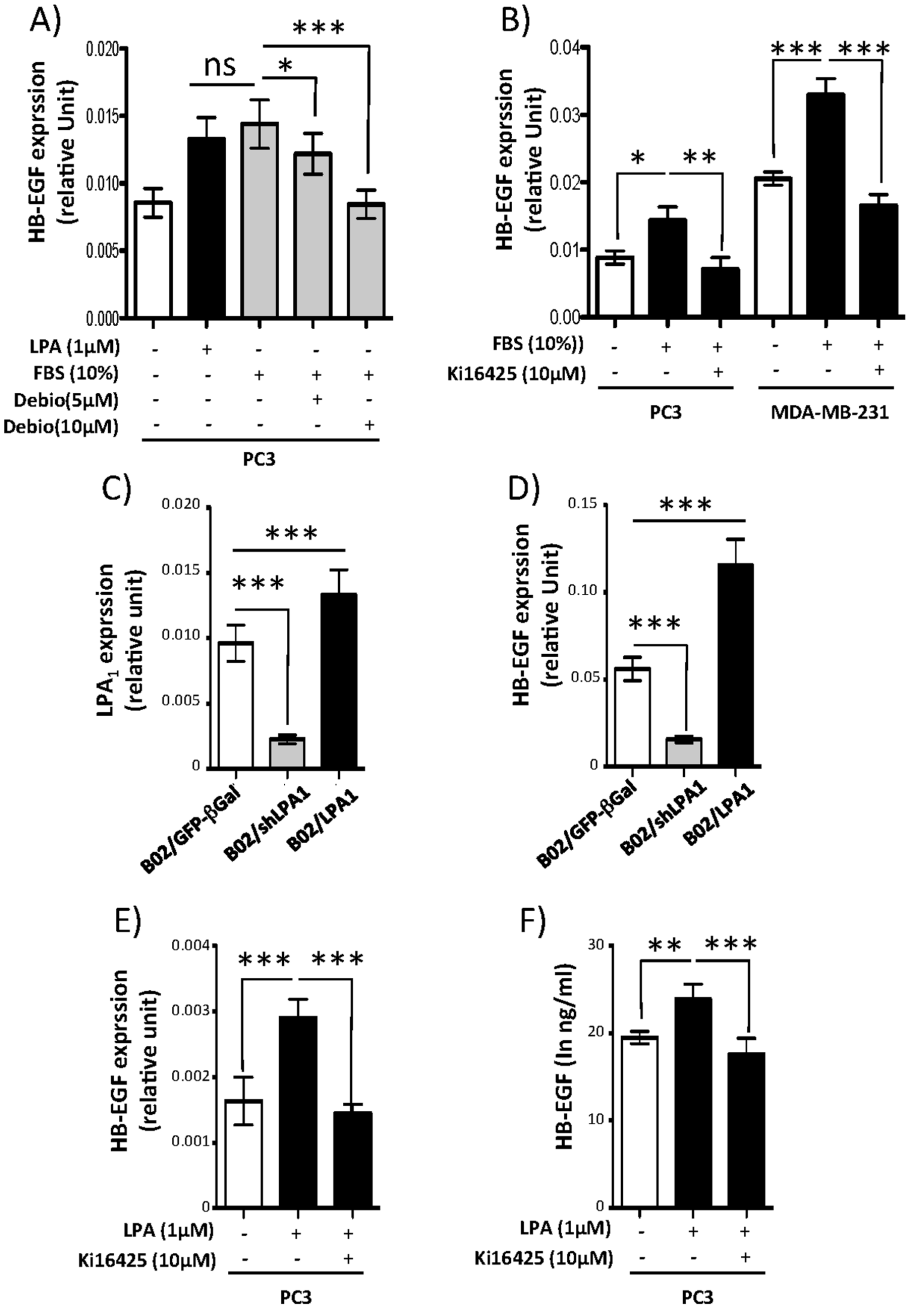
Expression of HB-EGF is mediated through functional LPA_1_ *in vitro*. (A,B,D,E) Expression of HB-EGF mRNA was measured by real-time quantitative PCR and normalized to housekeeping L32 gene in (A) PC3 cells treated for 45 min with LPA (1 µM) or fetal bovine serum (FBS, 10% w/v) in absence or presence of Debio0719 (Debio), (B) PC3 and MDA-MB-231 cells treated for 45 min with FBS (10% w/v) in absence or presence of Ki16425 (10 µM), (D) MDA-B02/GFP-βGal, MDA-B02/shLPA1 and MDA-B02/LPA1 cells culture in presence of FBS 10%, and (E) PC3 cells treated for 24 h with LPA (1 µM) in absence or presence of Ki16425 (10 µM). (C) Expression of LPA_1_ mRNA was measured by real-time quantitative PCR and normalized to housekeeping L32 gene in MDA-B02/GFP-βGal, MDA-B02/shLPA1 and MDA-B02/LPA1 cells cultured in the presence of 10% FBS. (F) Quantification of HB-EGF concentration in the conditioned culture media of PC3 cells treated for 24 h with LPA (1 µM) in absence or presence of Ki16425 (10 µM). All values were the mean±SD of at least three experiments. *p<0.05; **p<0.01; ***p<0.001 using one-way ANOVA with a Bonferroni post-test.

Among the 74 genes upregulated by LPA in a LPA_1_–dependent manner, 9 genes were coding for secreted proteins: 2 matrix proteins (COL27A1, COL6A1) and 7 cytokines and growth factors [IL6, IL-8, CSF2(GM-CSF), DTR(HB-EGF), CXCL1(Groα), CXCL2(Groβ), CXCL3(Groγ)]. Those genes were good candidates for additional analyses because of the potential use of their products as biomarkers detectable in biological fluids. We have described previously in the context of bone metastasis induced by breast cancer cells that LPA through LPA_1_ controls the expression of IL6, IL-8, CSF2(GM-CSF), and CXCL1(Groα) [18]. Here, we selected DTR coding for HB-EGF for further investigation because links between LPA_1_ and HB-EGF expression have not been described so far. We found that purified LPA (1 µM) was as potent as FBS (10%) in upregulating HB-EGF mRNA expression in PC3 cells after 45 min of stimulation (Figure 2A). The effect of FBS was totally blocked when cells were incubated in the presence of the LPA_1–3_ antagonist, Debio0719 [19]. This result was also observed in PC3 cells treated with another LPA_1–3_ antagonist, Ki16425 [21] (Figure 2B). Moreover, Ki16425 completely abolished FBS-induced expression of HB-EGF in MDA-MB-231 cells (Figure 2B). These cells do not express LPA_3_ (Figure 1A), supporting a prevalent role of LPA_1_ in HB-EGF expression. To confirm the functional implication of LPA_1_ in HB-EGF expression, we used three subclones of human MDA-B02 cells (MDA-B02/GFP-βGal, MDA-B02/LPA1, MDA-B02/shLPA1) that we generated in previous studies [18], [27], [28], [31]. We first validated by RT-QPCR the expression levels of LPA_1_ in these cells. As expected, MDA-B02/GFP- β Gal cells express LPA_1_ that is stably overexpressed in MDA-B02/LPA1 cells and stably downregulated in MDA-B02/shLPA1 cells (Figure 2C). RT-QCR analyses on these cells cultured in the presence of FBS (10%) showed that expression of HB-EGF was significantly higher in MDA-B02/LPA1 cells and lower in MDA-B02/shLPA1 cells than in MDA-B02/GFP-βGal cells, respectively, confirming the direct control of HB-EGF expression through LPA_1_ activation (Figure 2D). We then tested the capacity of LPA to induce the expression of HB-EGF after a more extended time of stimulation. PC3 cells were treated for 24 h with LPA. Cells were harvested for RNA extraction and mRNA quantifications, and culture media were collected for secreted protein analysis. We observed that LPA treatment upregulated the expression of HB-EGF mRNA (Figure 2E) and the secretion of the protein (Figure 2F) by PC3 cells. Both LPA-induced upregulation of HB-EGF mRNA and protein secretion were totally inhibited in the presence of Ki16425 (Figures 2E, 2F).

### Expression of HB-EGF correlates with LPA_1_ expression in human primary tumors of breast, prostate, lung and colon cancers

**Figure 3:**
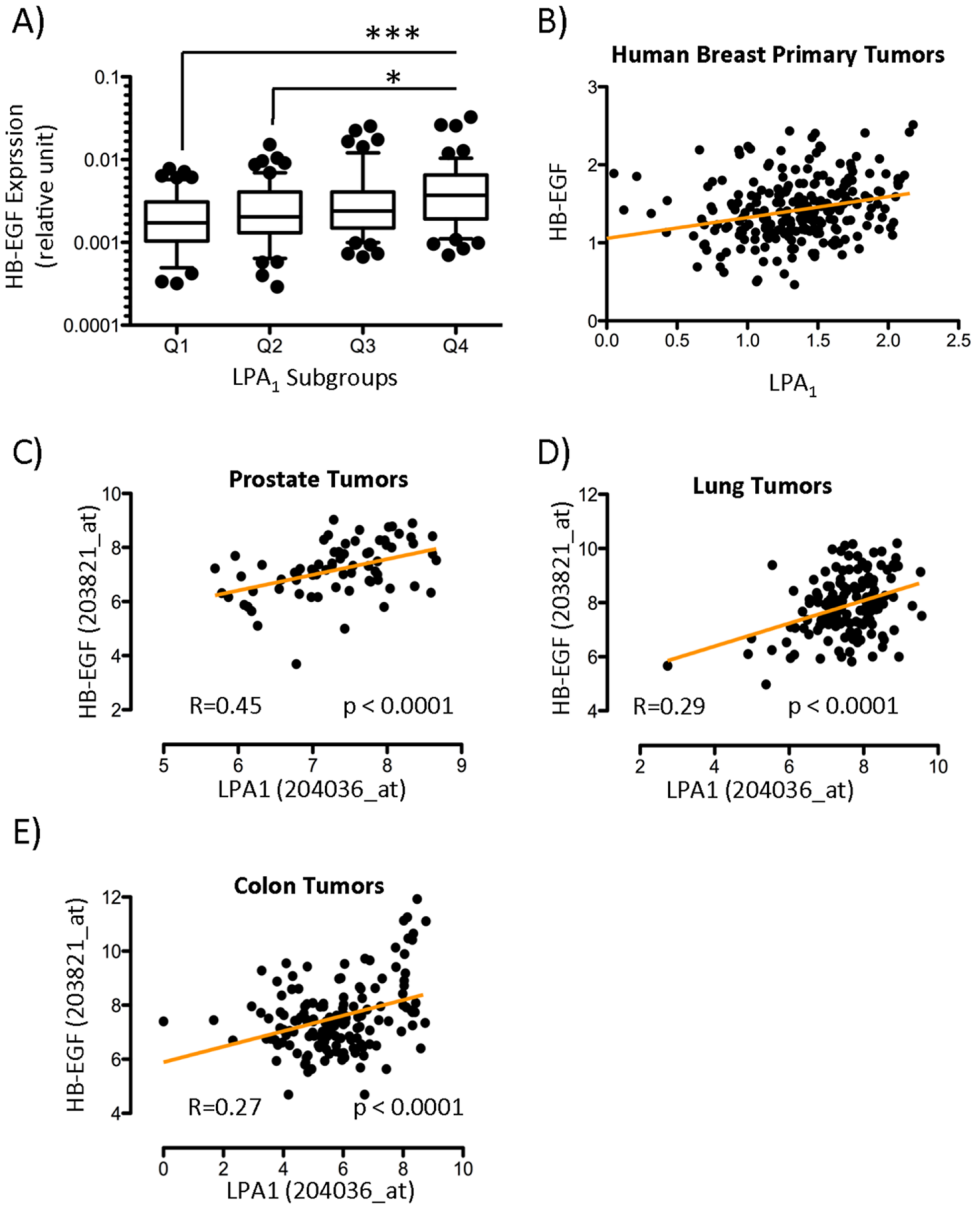
Increased expression of HB-EGF linked to high expression of LPA_1_ in human primary tumors of breast, prostate, lung and colon. (A) Total RNAs were extracted from 234 human primary breast tumor biopsies. Expression of LPA_1_ mRNA was measured by real-time quantitative PCR and normalized to housekeeping L32 gene values. LPA_1_ relative expression values were distributed into quartiles (Q) dividing the 234 primary tumors into four equal groups with equal frequencies. HB-EGF relative expression values in each LPA_1_ subgroups were represented in box plot. All values are the mean±SD of each quartile. *p<0.05; ***p<0.001 vs. Q4 using Kruskal-Wallis with Dunn's post-test. (B) Scatter plot was constructed showing the correlation between LPA_1_ and HB-EGF (R Spearman = 0.25; *p*<0.0001) in the same RT-QPCR data. Scatter plots of LPA_1_ and HB-EGF expression were constructed with the Log2 tranformed values extracted from publically available databases using R2 genomics analysis and visualization platform for (C) Prostate Tumor (GSE2109; n = 72; R Spearman = 0.45; *p*<0.0001); (D) Lung Tumor (GSE43580; n = 150; R Spearman = 0.29; *p*<0.0001) and (E) Colon Tumor (GSE21510; n = 148; R Spearman = 0.27; *p*<0.0001).

We then asked if the activity of LPA through LPA_1_ inducing HB-EGF expression observed in our breast cancer cell lines could be also observed in clinical human samples. We analyzed the expression LPA_1_ and HB-EGF by real-time quantitative PCR in a series of 234 primary tumor biopsies from patients with breast cancer. We then distributed tumors form patients into four quartiles according to the expression values of LPA_1_ (Q1, Q2, Q3, Q4) and reported the mean relative expression value of HB-EGF in each group. We observed that the LPA_1_-Q4 group, which has the highest level of LPA_1_ expression, has a significantly higher expression of HB-EGF than groups with lower expression of LPA_1_ (Q1 and Q2) (Figure 3A). We also observed that there was a statistically significant positive correlation between LPA_1_ and HB-EGF in these samples (Figure 3B). To further confirm our findings, we then looked into publically available databases for Prostate Tumor, Lung Tumor, and Colon Tumor. We observed that in the prostate tumor database there was a moderate but highly statistically significant positive correlation between LPA_1_ and HB-EGF (Figure 3C). In addition, in lung and colon tumor databases we observed a low but still highly statistically significant positive correlation between these two genes (Figure 3D and 3E). Therefore, this result supports the hypothesis for a positive correlation between LPA_1_ expression and HB-EGF expression *in vivo*.

### Pharmacological blockade of LPA_1_ in vivo inhibits HB-EGF secretion by human PC3 xenographs

**Figure 4:**
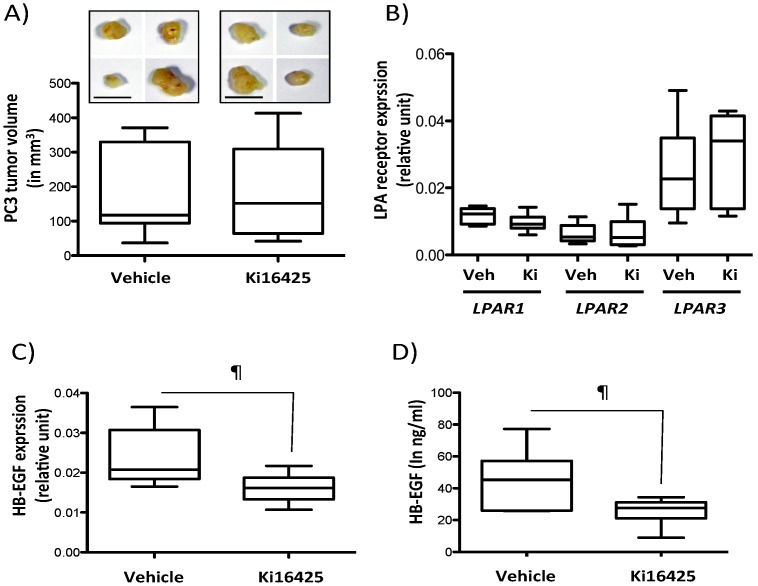
Pharmacological blockade of LPA_1_ *in vivo* inhibits HB-EGF secretion by human PC3 xenographs. PC3 tumor cells were injected subcutaneously in the right flank of male BALB/C nude mice. At day 35, post-tumor cell injection animals were randomized into two groups and treated with Ki16425 (25 mg/kg) or the vehicle for 5 d. (A) Representative photographs of primary tumors at day 40 (upper panels). Box plot represent tumor volumes (in mm^3^) (lower panels). Bar represents 10 mm. (B) *LPAR1*, *LPAR2* and *LPAR3* expressions were measured by real-time quantitative PCR and normalized to housekeeping *L32* gene values (Veh: Vehicle; Ki: Ki16425) (C) Box plot represents expression of HB-EGF mRNA expression detected by real-time quantitative PCR from total RNAs isolated from tumors of animals treated with vehicle or Ki16425. Values were normalized to housekeeping L32 gene. ¶: *p*<0.05, using unpaired Student t-Test. (D) Box plot represents HB-EGF concentration detected by ELISA in the serum of animals treated with Ki16425 or vehicle. ¶: *p*<0.05, using unpaired Student t-Test.

To confirm if LPA_1_ activity may control HB-EGF expression *in vivo*, we generated xenograph tumors by subcutaneous injection of PC3 cells in the right flank of male BALB/C nude mice. After 35 days, post-tumor cell injection animals were treated subcutaneously with Ki16425 (25 mg/kg) for 5 d. Animals were then sacrificed. At that time, we collected the tumors for RNA preparation and the serum of animals for protein quantification. First, we found that a systemic treatment of animals for five days with the LPA_1_ blocker did not alter tumor growth as judged by the absence of difference in the volume of PC3 tumors in animals treated with Ki16425 compared to vehicle-treated mice (Figure 4A). This result was in agreement with previous reports of our laboratory and others using breast cancer mouse models [19], [20]. We also found that Ki16425 treatment did not affect the expression levels of LPA receptors in these tumors (Figure 4B). However, we found that the level of HB-EGF mRNA was significantly decreased by 28% in tumors of animals treated with Ki16425 compared to tumors of animals treated with the vehicle (*p* = 0.0231; 0.0169±0.0011 vs. 0.02327±0.0028) (Figure 4C). Moreover, we observed that the concentration of human HB-EGF was significantly decreased by 44% in the serum of animals treated with Ki16425 compared to that of animals treated with the vehicle (*p* = 0.0321; 25.35±3.14 vs. 44.93±6.87) (Figure 4D). These results indicate that blocking LPA_1_ activity *in vivo* inhibits the expression and secretion of HB-EGF in animals bearing PC3 xenograph tumors.

## Discussion

Studies using genetically modified cells and mice have revealed the functional involvements of LPA_1_ in multiple pathological processes. Overexpression of *Lpar1* driven by the MMTV promoter in the mammary gland of transgenic mice induces the formation of spontaneous breast tumors within the first year [17]. Using immune compromised mice we showed that expression of LPA_1_ confers a high propensity of inducing bone metastasis to human breast cancer cells [18]. In that context, treatments with the LPA_1–3_ antagonist Ki16425 inhibited the progression of osteolytic bone metastases [18]. In addition, we recently described that the LPA_1–3_ antagonist Debio0719 prevented spontaneous metastasis dissemination of mouse 4T1 mammary cancer cells to bone and lungs [19] with additional induction of tumor cell dormancy at secondary sites [20]. However, validating that LPA_1–3_ antagonists were inhibiting LPA_1_-specific pro-oncogenic and pro-metastatic activities could not be achieved in these studies because of missing LPA_1_-specific biomarkers.

Our transcriptomic analysis confirmed previous findings showing that LPA induces the expression of the pro-osteoclastic cytokines IL6 and IL8 through LPA_1_ in breast cancer cells [18], [28]. The capacity of LPA_1_ in inducing the secretion of these cytokines was further extended to ovarian cancer cells [23]. LPA_1_ shares this activity with other LPA receptors as LPA_3_ also mediates both IL6 and IL8 expressions and LPA_2_ was identified to be the most efficient receptor in linking LPA to IL-6 and IL-8 production in ovarian cancer cells [24]. Therefore, IL6 and IL8 are unlikely relevant LPA_1_-specific biomarkers. Among other cytokines, LPA induces the expression of CYR61 and CTGF in epithelial cells [25], [32]–[34]. Analyses of *Lpar1* ^−/−^ animals or pharmacological blockade of LPA receptors with Ki16425 revealed a major role of LPA_1_ in LPA-induced CYR61 and CTG expressions in bacterial infection and induction of tissue fibrosis in kidney and lungs [25], [32]–[34]. We observed that LPA induced the expression of both CYR61 and CTGF in prostate and breast cancer cells (data not shown). Expression of CYR61 and CTGF in MDA-MB-231 and MCF-7 cells were previously reported, corroborating that expression of these two cytokines are not restrained to LPA_1_-expressing cells [35]. CYR61 and CTGF genes are also upregulated by TGF-β and commonly found in TGF-β gene signatures in different cellular models [26], [36], [37]. Recent reports showed that LPA-dependent expression of CTGF requires transactivation of TGF-β receptor in myoblasts [38]. Thus, the specificity of CTGF expression through LPA_1_ activation requires further demonstration. Based on cross-comparison analyses of whole genome transcriptomic profiles of PC3, MDA-MB-231 and MCF-7 cells in response to LPA, we determined a list of early genes upregulated through LPA_1_ activation. In the perspective of establishing a new biomarker easily accessible for sample examination in clinic, we focused our study on the secreted growth factor HB-EGF. We found that HB-EGF expression was detected at an early time point following LPA stimulation (45 min). Its expression was sustained at least up to 24 h of stimulation *in vitro* and 5 d *in vivo*, suggesting the potential use of HB-EGF as a relevant marker in the perspective of mid- to long-term follow-up. Based on the quantification of HB-EGF *in vitro*, our results demonstrated that the treatment of MDA-MB-231 breast and PC3 prostate cancer cells with LPA_1–3_ antagonists (Ki16425, Debio0719) mimicked *Lpar1* gene silencing in MDA-B02 breast cancer cells. Also, we found a significantly higher expression of HB-EGF mRNA in human primary breast tumors that highly expressed LPA_1_. Unfortunately, we were not able to confirm this correlation at the protein level because biological samples from patients of this cohort were not available. However, we were able to quantify by ELISA the concentration of human HB-EGF secreted by PC3 cells in the blood circulation of PC3 tumor-bearing animals. Moreover, we validated that the pharmacological blockade of LPA_1_ can be monitored *in vivo* through the inhibition of HB-EGF expression both at mRNA levels in the primary tumors and at protein levels in the blood circulation. LPA was previously shown inducing the shedding of proHB-EGF in VeroH cells through a Ras-Raf-MEK-dependent pathway [39]. LPA also induces the shedding of proHB-EGF from the membrane of in human bronchial epithelial cells involving activation of matrix metalloproteinases leading to down-stream stimulation of EGFR and the secretion of IL-8 [40]. However, these works did not characterize which LPA receptor transmitted LPA signals. Our results demonstrated at mRNA and protein levels that LPA induces HB-EGF expression through an LPA_1_-dependent mechanism. Our study was based on manipulating breast and prostate cancer cells and on analyzing publically available tumor databases of breast, prostate, lung and colon cancers. Therefore, the significance of HB-EGF as a biomarker of LPA_1_ activity in other pathologies, such as tissue fibrosis [14], [25], obesity [41], rheumatoid arthritis [15], osteoporosis [5], and neuropathic pain [42], remains to be determined.

Disease-free and overall survivals are frequent primary endpoints validating drug efficacies in clinical trials. However, using intermediate endpoints and biomarkers are required during the course of therapies to validate drugs having reached their dedicated targets. Our findings revealed that HB-EGF is a new biomarker downstream stimulation of LPA_1_ that would be extremely valuable to quantify the LPA_1_ activation state in patients receiving anti-LPA_1_ therapies.
